# Supplementary material for: Assessing the inter- & intra-reliability of a customised volleyball performance analysis system to analyse complexes and the efficacy of the associated skills
Source: PLoS One. 2025 Nov 26;20(11):e0337579. doi: 10.1371/journal.pone.0337579 (PMC12654878; doi:10.1371/journal.pone.0337579)
Supplement: S1 Table — (DOCX) [file pone.0337579.s001.docx]

**Definitions for Serve and Type of Serve.**

| **Skill/Technique** | | | **Definition** | | | | | | | **Abbreviation** | | |  |
| --- | --- | --- | --- | --- | --- | --- | --- | --- | --- | --- | --- | --- | --- |
| *Serve* | | | *This skill starts the rally. The player serves the ball from behind the back line of the court. If their team wins the rally the player serves again, they will continue until they lose the rally when the serve will go to the other team.* | | | | | | | *SV* | | |  |
|  |  |  |  |  |  |  |  |  |  |  |  |  |  |
|  |  |  |  |  |  |  |  |  |  |  |  |  |  |
|  |  |  |  |  |  |  |  |  |  |  |  |  |  |
|  |  |  |  |  |  |  |  |  |  |  |  |  |  |
| Power Jump Serve | | | This type of serve requires the player to perform a three-step approach whilst the ball is in the air before contacting it. The velocity of the ball is higher than that of a float serve, and during its flight the ball spins. | | | | | | | PJ | | |  |
|  |  |  |  |  |  |  |  |  |  |  |  |  |  |
|  |  |  |  |  |  |  |  |  |  |  |  |  |  |
|  |  |  |  |  |  |  |  |  |  |  |  |  |  |
| Standing Power Serve | | | This type of serve is similar to the jump variant however, it does not require the player to jump when contacting the ball. | | | | | | | PS | | |  |
|  |  |  |  |  |  |  |  |  |  |  |  |  |  |
|  |  |  |  |  |  |  |  |  |  |  |  |  |  |
| Float Jump Serve | | | This type of serve requires the player to perform a three-step approach whilst the ball is in the air before contacting it. The velocity of the ball is lower than a power serve, but the ball moves in the air making it more difficult for the defence to read. | | | | | | | FJ | | |  |
|  |  |  |  |  |  |  |  |  |  |  |  |  |  |
|  |  |  |  |  |  |  |  |  |  |  |  |  |  |
|  |  |  |  |  |  |  |  |  |  |  |  |  |  |
|  |  |  |  |  |  |  |  |  |  |  |  |  |  |
| Standing Float Serve | | | This type of serve is similar to the jump variant however, it does not require the player to jump when contacting the ball. | | | | | | | FS | | |  |
