# Supplementary material for: Assessing the inter- & intra-reliability of a customised volleyball performance analysis system to analyse complexes and the efficacy of the associated skills
Source: PLoS One. 2025 Nov 26;20(11):e0337579. doi: 10.1371/journal.pone.0337579 (PMC12654878; doi:10.1371/journal.pone.0337579)
Supplement: S2 Table — (DOCX) [file pone.0337579.s002.docx]

**Definitions of Spike and Type of Spike**

| **Skill/Technique** | | | **Definition** | | | | | | | **Abbreviation** | | |  |
| --- | --- | --- | --- | --- | --- | --- | --- | --- | --- | --- | --- | --- | --- |
| *Spike* | | | *The spike typically ends the phase of play/complex. The player performing it is looking to hit the ball on a steep trajectory so that it lands within the court boundaries and can't be defended by the opposition.* | | | | | | | *SP* | | |  |
|  |  |  |  |  |  |  |  |  |  |  |  |  |  |
|  |  |  |  |  |  |  |  |  |  |  |  |  |  |
|  |  |  |  |  |  |  |  |  |  |  |  |  |  |
|  |  |  |  |  |  |  |  |  |  |  |  |  |  |
| Spike Line | | | A spike that is referred to as being 'line' refers to it being directed, ahead of the hitter on the oppositions back court, and crosses the 3m line. | | | | | | | SPL | | |  |
|  |  |  |  |  |  |  |  |  |  |  |  |  |  |
|  |  |  |  |  |  |  |  |  |  |  |  |  |  |
| Spike Diagonal | | | Also referred to 'cross-court', this spike follows a diagonal trajectory (e.g. an outside hitter will spike towards the back right corner, or the opposite will spike towards the back left corner), and crosses the 3m line. A Middle Blocker is able to hit towards both back corners due to their position on the front court. | | | | | | | SPD | | |  |
|  |  |  |  |  |  |  |  |  |  |  |  |  |  |
|  |  |  |  |  |  |  |  |  |  |  |  |  |  |
|  |  |  |  |  |  |  |  |  |  |  |  |  |  |
|  |  |  |  |  |  |  |  |  |  |  |  |  |  |
|  |  |  |  |  |  |  |  |  |  |  |  |  |  |
| Spike Swipe | | | This spike technique is different to the previous two techniques discussed. As opposed to looking to ground the ball, the hitter is seeking to swipe the ball off the opposition block and out of play. Typically landing in the front court. | | | | | | | SPS | | |  |
|  |  |  |  |  |  |  |  |  |  |  |  |  |  |
|  |  |  |  |  |  |  |  |  |  |  |  |  |  |
|  |  |  |  |  |  |  |  |  |  |  |  |  |  |
| Spike Tip | | | Similar to the Spike Swipe, a tip looks to use the block to win the point without putting a great velocity on the ball. Rather the aim is to get the the ball to go over the block and land just behind it negating the defence that will have stepped back expecting a line or diagonal spike. Landing in the front court. | | | | | | | SPT | | |  |
