# Supplementary material for: Assessing the inter- & intra-reliability of a customised volleyball performance analysis system to analyse complexes and the efficacy of the associated skills
Source: PLoS One. 2025 Nov 26;20(11):e0337579. doi: 10.1371/journal.pone.0337579 (PMC12654878; doi:10.1371/journal.pone.0337579)
Supplement: S3 Table — (DOCX) [file pone.0337579.s003.docx]

**Definitions of Block and Type of Block.**

| **Skill/Technique** | | | **Definition** | | | | | | | **Abbreviation** | | |  |
| --- | --- | --- | --- | --- | --- | --- | --- | --- | --- | --- | --- | --- | --- |
| *Block* | | | *The block is the first defensive action a team can perform (apart from when receiving the serve). Players on the front court will look to jump and raise their hands above the height of the net in front of the hitter. They are aiming to force the hitter to aim towards a player with a strong defence or to contact the ball.* | | | | | | | *B* | | |  |
|  |  |  |  |  |  |  |  |  |  |  |  |  |  |
|  |  |  |  |  |  |  |  |  |  |  |  |  |  |
|  |  |  |  |  |  |  |  |  |  |  |  |  |  |
|  |  |  |  |  |  |  |  |  |  |  |  |  |  |
|  |  |  |  |  |  |  |  |  |  |  |  |  |  |
|  |  |  |  |  |  |  |  |  |  |  |  |  |  |
| 3 Person | | | The approach to blocking also has all three players attempting to block the hitter. | | | | | | | B3P | | |  |
|  |  |  |  |  |  |  |  |  |  |  |  |  |  |
|  |  |  |  |  |  |  |  |  |  |  |  |  |  |
| 2 Person | | | The approach to blocking also has two players attempting to block the hitter. | | | | | | | B2P | | |  |
|  |  |  |  |  |  |  |  |  |  |  |  |  |  |
|  |  |  |  |  |  |  |  |  |  |  |  |  |  |
| 1 Person Block | | | This blocking approach is when a player is isolated at the front court against a hitter and performs the block by themselves. | | | | | | | B1P | | |  |
