# Supplementary material for: Assessing the inter- & intra-reliability of a customised volleyball performance analysis system to analyse complexes and the efficacy of the associated skills
Source: PLoS One. 2025 Nov 26;20(11):e0337579. doi: 10.1371/journal.pone.0337579 (PMC12654878; doi:10.1371/journal.pone.0337579)
Supplement: S4 Table — (DOCX) [file pone.0337579.s004.docx]

**Definitions of Serve Receive and Type of Serve Receive.**

| **Skill/Technique** | | | **Definition** | | | | | | | **Abbreviation** | | |  |
| --- | --- | --- | --- | --- | --- | --- | --- | --- | --- | --- | --- | --- | --- |
| *Serve Receive* | | | *This is the first contact of the ball following the serve. The player performing this is seeking to get the ball to the setter in a position that provides the most attacking options.* | | | | | | | *SR* | | |  |
|  |  |  |  |  |  |  |  |  |  |  |  |  |  |
|  |  |  |  |  |  |  |  |  |  |  |  |  |  |
|  |  |  |  |  |  |  |  |  |  |  |  |  |  |
| Serve Receive Underhand | | | The underarm technique is commonly referred to as a dig technique, whereby the player creates a platform with their forearms to direct the ball to the setter. | | | | | | | SRU | | |  |
|  |  |  |  |  |  |  |  |  |  |  |  |  |  |
|  |  |  |  |  |  |  |  |  |  |  |  |  |  |
|  |  |  |  |  |  |  |  |  |  |  |  |  |  |
| Serve Receive Overhand | | | The overhand technique, traditionally, refers to a volley often used against slower serves (i.e. float serves), but can also include different variants that have entered the game from beach volleyball. | | | | | | | SRO | | |  |
|  |  |  |  |  |  |  |  |  |  |  |  |  |  |
|  |  |  |  |  |  |  |  |  |  |  |  |  |  |
