# Supplementary material for: Assessing the inter- & intra-reliability of a customised volleyball performance analysis system to analyse complexes and the efficacy of the associated skills
Source: PLoS One. 2025 Nov 26;20(11):e0337579. doi: 10.1371/journal.pone.0337579 (PMC12654878; doi:10.1371/journal.pone.0337579)
Supplement: S5 Table — (DOCX) [file pone.0337579.s005.docx]

**Definitions of Set and Type of Set.**

| **Skill/Technique** | | | **Definition** | | | | | | | **Abbreviation** | | |  |
| --- | --- | --- | --- | --- | --- | --- | --- | --- | --- | --- | --- | --- | --- |
| *Set* | | | *The set is traditionally an overhand technique performed by the setter on the 2nd contact. The intention of the set is to provide the attacker with the best opportunity to spike the ball and win the point. Should the play be 'out of system' then other players may set the ball rather than the setter.* | | | | | | | *S* | | |  |
|  |  |  |  |  |  |  |  |  |  |  |  |  |  |
|  |  |  |  |  |  |  |  |  |  |  |  |  |  |
|  |  |  |  |  |  |  |  |  |  |  |  |  |  |
|  |  |  |  |  |  |  |  |  |  |  |  |  |  |
|  |  |  |  |  |  |  |  |  |  |  |  |  |  |
| Tempo 1 | | | Often referred to by coaches and players as a 'shoot' (for outsides and opposites) or a 'quick' (for middle blockers). This is the quickest velocity of the set, which has a flatter trajectory. | | | | | | | T1 | | |  |
|  |  |  |  |  |  |  |  |  |  |  |  |  |  |
|  |  |  |  |  |  |  |  |  |  |  |  |  |  |
|  |  |  |  |  |  |  |  |  |  |  |  |  |  |
| Tempo 2 | | | This is the traditional velocity of a set with more of an arc to the flight of the ball, especially when setting the outside and opposites. For middle blockers, the distance the ball travels will be less, but the trajectory of the ball will be higher. | | | | | | | T2 | | |  |
|  |  |  |  |  |  |  |  |  |  |  |  |  |  |
|  |  |  |  |  |  |  |  |  |  |  |  |  |  |
|  |  |  |  |  |  |  |  |  |  |  |  |  |  |
|  |  |  |  |  |  |  |  |  |  |  |  |  |  |
| Tempo 3 | | | This set applies a slower velocity to the ball, often used when having to set the ball across longer distances towards the Outside Hitter or Opposite, or when not set by the Setter. | | | | | | | T3 | | |  |
|  |  |  |  |  |  |  |  |  |  |  |  |  |  |
|  |  |  |  |  |  |  |  |  |  |  |  |  |  |
|  |  |  |  |  |  |  |  |  |  |  |  |  |  |
| Setter Dump | | | The Setter Dump is an attacking play used when they are in a front row rotation. They will fake a set but push the ball onto the opposition's court, aiming to win the point. | | | | | | | 2nd C | | |  |
