# Supplementary material for: Assessing the inter- & intra-reliability of a customised volleyball performance analysis system to analyse complexes and the efficacy of the associated skills
Source: PLoS One. 2025 Nov 26;20(11):e0337579. doi: 10.1371/journal.pone.0337579 (PMC12654878; doi:10.1371/journal.pone.0337579)
Supplement: S6 Table — (DOCX) [file pone.0337579.s006.docx]

**Definitions of Defence and Type of Defence.**

| **Skill/Technique** | | | **Definition** | | | | | | | **Abbreviation** | | |  |
| --- | --- | --- | --- | --- | --- | --- | --- | --- | --- | --- | --- | --- | --- |
| *Defence* | | | *This is typically the first contact of the ball being returned from an attack, block, or freeball. The player performing this is seeking to get the ball to the setter in a position that provides the most attacking options. It may appear more than once if a team can’t construct an attacking opportunity.* | | | | | | | *D* | | |  |
|  |  |  |  |  |  |  |  |  |  |  |  |  |  |
|  |  |  |  |  |  |  |  |  |  |  |  |  |  |
|  |  |  |  |  |  |  |  |  |  |  |  |  |  |
| Defence Underhand | | | The underarm technique is commonly referred to as a dig technique, whereby the player creates a platform with their forearms in order to direct the ball to the setter. | | | | | | | DO | | |  |
|  |  |  |  |  |  |  |  |  |  |  |  |  |  |
|  |  |  |  |  |  |  |  |  |  |  |  |  |  |
|  |  |  |  |  |  |  |  |  |  |  |  |  |  |
| Defence Overhand | | | The overhand technique, traditionally, refers to a volley often used against slower attacks (i.e. tips) but can also include different variants that have entered the game from beach volleyball. | | | | | | | DO | | |  |
|  |  |  |  |  |  |  |  |  |  |  |  |  |  |
|  |  |  |  |  |  |  |  |  |  |  |  |  |  |
