# Supplementary material for: Assessing the inter- & intra-reliability of a customised volleyball performance analysis system to analyse complexes and the efficacy of the associated skills
Source: PLoS One. 2025 Nov 26;20(11):e0337579. doi: 10.1371/journal.pone.0337579 (PMC12654878; doi:10.1371/journal.pone.0337579)
Supplement: Attachment — Submitted filename: Review.pdf [file pone.0337579.s007.pdf]

Review

This report tries to be critically constructive so that a research paper to test the inter-observer and intra-observer reliability of a specific performance analysis system, i.e., in terms of analysing the complexes and in terms of the accompanying skill efficacy, can be strengthened. While the manuscript has reported interesting preliminary results, and the testing methodology of the reliability was described and wholly scientific, there are a few overarching areas that have scope for considerable improvement, specifically in terms of methodological description and nomenclatural correctness.

The proposed five-level efficacy scale requires significant reconceptualisation to align it with contemporary volleyball performance analysis requirements. In today's analysis, the assessment of skill is based on the product created rather than the quality of movement per se. A guiding principle, as conceptualised by Coleman et al. (1971), dictates that the measurement of one team's serve must have an inversely related relationship with the opposing team's receive of the serve. The principle is not uniformly applied in the current scale.

More methodologically sound for assessing serve and serve reception would be to employ a reverse evaluation structure with a small degree change to ensure mutual exclusivity and logical sequence:

1. Serve Reception Modification: To eliminate the occurrence of a serve reception followed by a serve fault (Level 0), the serve reception scale must be truncated or shifted at least one level of efficacy. The remaining levels must be logically reversed with respect to serve efficacy levels (e.g., Level 1 Serve ↔ Level 4 Serve Reception; Level 2 Serve ↔ Level 3 Serve Reception, etc.).

2. Increased Differentiation in Serve Reception: Leaning on the precision of spatiotemporal recording systems like Nacsport Scout, the range for the non-scoring action (serve reception) should be expanded to more effectively distinguish among game situations created by the serve. Specifically, the lowest effective level of reception (presumably including "ball hit straight back to server" or "passed out from setter") should be separated into two distinct efficacy levels: Level 1: Ball is returned back directly to the serving team, with the potential to set up a counterattack (the receiving team must defend) and Level 2: Ball is served away from the setter, setting up a possible out-of-system attack (the receiving team still retains the right to attack). This is a significant distinction, as the following game dynamics are indeed different and need different efficacy ratings.

3. Serve Scale Discrimination: Level 3 of the serve scale, which is currently combining an out-of-system attack for the receiving team and a freeball for the serving team, must be fortified. Within competitive volleyball, the two scenarios are opposed opportunities for the serving team and must be seized by different assessment levels.

Block Evaluation Scale Clarity

The Block Evaluation Scale must contain greater definitional clarity across a number of different levels:

• Level 0 (Error/Point): Authors should specify the nature of error clearly. It needs clarification if Level 0 involves a blocking player's fault at the net or a block that causes the ball to touch the attacking side and subsequently fly out of the court.

• Level 1 (Clear Block): The definition of "clear block" ought to explicitly define its role as a known tactic (especially in women's volleyball) to intentionally guide the attack route towards a specific, specialised defence area or player.

•Level 3 (Block with Ball Remaining on Attacking Side): Improve this level. When a block has taken place, and the ball remains on the attacking side, the right to attack still exists for the team. This is a good plus, even though the subsequent first ball contact is not ideal, and the evaluation scale must reflect this tactical plus.

<u>Setting Evaluation</u>

The current Setting evaluation scale presents formidable practical challenges to observers, even at the expense of system reliability. It necessitates setting a set as "in the general area of the hitter" to require the observer to judge simultaneously the spatiotemporal relationship between two dynamically moving points: the trajectory and appropriate speed of the ball, and the attacking centre of mass jumping (diagonal, vertical, or lateral movement). This very high degree of observer judgment provides the likelihood of measurement error and must be addressed either by the provision of more objective, quantifiable criteria or through the simplification of level definition. In my opinion, the evaluation of setting skill demands a more complex approach for the observer. Please see Fellingham et al. (2013); Florence et al. (2008).

<u>Skill Definition Requirements</u>

The manuscript lacks the operational definitions required for all skill types. These are the precursors to reproducibility and reliability.

•Spike/Attack Terminology: Spike classification is taxonomically inconsistent. Diagonal and line spikes are direction-based categories, while swipe and tip (or dink) are contact technique-based categories. A swipe, for instance, can be executed as a diagonal or line attack. The authors must use a consistent classification criterion.

• Tempo Setting: Clear and unambiguous definitions for setting tempos need to be established, utilizing definite, established literature (Afonso et al., 2010) to ensure external validity and consistency with prior work in performance analysis.

<u>Methodological Deficiencies and Efficacy Assessment</u>

The study's stated objective was "to examine…, position,... and skill efficacy."

The Methods section is inadequate because it provides no traceable information regarding the variable of position (e.g., player position, ball position, or rotation scheme by the team).

Moreover, the paper does not include the mathematical formula or equation used in calculating the efficacy scores of skills, a necessary prerequisite to validate any bespoke performance analysis system. Not including this aspect makes it difficult for the peer reviewer and subsequent researchers to identify or reproduce the efficacy measure.

Dear Authors,

I have carefully reviewed your work and appreciate your efforts to contribute to this field of research. While your research topic is of interest and has potential, I regret to inform you that I'm unable to propose acceptance of your manuscript in its current form. As the efficacy scale of volleyball skills was a basic instrument for the research, several methodological shortcomings and limitations were identified that require substantial revisions to ensure the paper's scientific validity. I believe that with careful attention to these areas, your manuscript has the potential to make a valuable contribution to the field. I would like to encourage you to revise your work and resubmit it for consideration.

Bibliography

Afonso, J., Mesquita, I., Marcelino, R., & António Da Silva, J. (2010). Analysis of the setter's tactical action in high-performance women's volleyball. *Kinesiology*, *42*, 82–89.

Coleman, J. E., Neville, B., & Gordon, B. (1971). A statistical system for volleyball its use in Chicago women's association. *International Volleyball Review*, *27*, 72–73.

Fellingham, G. W., Hinkle, L. J., & Hunter, I. (2013). Importance of attack speed in volleyball. *Journal of Quantitative Analysis in Sports*, *9*(1), 87–96. https://doi.org/10.1515/JQAS-2012-0049

Florence, L. W., Fellingham, G. W., Vehrs, P. R., & Mortensen, N. P. (2008). Skill Evaluation in Women's Volleyball. *Journal of Quantitative Analysis in Sports*, *4*(2), 1–16. https://doi.org/10.2202/1559-0410.1105
