# Supplementary material for: Assessing the inter- & intra-reliability of a customised volleyball performance analysis system to analyse complexes and the efficacy of the associated skills
Source: PLoS One. 2025 Nov 26;20(11):e0337579. doi: 10.1371/journal.pone.0337579 (PMC12654878; doi:10.1371/journal.pone.0337579)
Supplement: Attachment — Submitted filename: Reliability Study; Repsonse to reviewers.docx [file pone.0337579.s009.docx]

Firstly, thank you for your time and efforts reviewing this paper, it is greatly appreciated. Your comments have helped in developing and improving the paper. We hope that the changes and responses address the points raised.

Any changes have been highlighted in yellow throughout the amended manuscript.

**General Feedback**

Please include a copy of Tables 5, 6, 7, 8, which you refer to in your text on page 17.

Apologies, this was a labelling issue and has since been corrected. Page 17. Lines 262 and 266-267

**Responses to Reviewer 1**

This study presents a thorough investigation into the inter and intrareliability of a bespoke volleyball notational analysis system that incorporates complexes, player position, skill type, and skill efficacy. The research addresses a significant gap in the performance analysis literature b not only assessing reliability for a complex set of variables but also by explicitly monitoring for observer drift over an extended period (nine months). The methodological approach is robust, with a detailed description of the coder training process, appropriate statistical analyses (Cohen's Kappa, Weighted Kappa, ICC), and a substantial dataset. The findings are valuable for researchers and practitioners in volleyball performance analysis, demonstrating that detailed, multifaceted coding systems can achieve high reliability. The manuscript is well structured and clearly written. I recommend Minor Revisions prior to publication.

Thank you for taking the time to review our manuscript. We are pleased that you are able to see the value in the work completed and its application to practise and research. In responding to your useful comments and suggestions, we have refined the manuscript, providing further clarity regarding our findings and limitations.

The current statement indicates that data cannot be shared due to an agreement with a professional volleyball organization. While this is understandable, it would be beneficial to clarify if there are any conditions under which the data could be made available (e.g., upon reasonable request to the corresponding author and with permission from the organization) or if a deidentified/minimal dataset could be provided. As per PLOS ONE policy, simply stating data is available on request is insufficient, but a more detailed justification for restricted access is needed.

We appreciate your understanding of the situation regarding the data sharing agreement in place. Upon review and further discussion, we have decided to make the data set available through the institutional repository (PURE), but this can only be uploaded following confirmation of acceptance. We initially sought to not share the data set and then considered reasonable request, due to the position of this study, the associated data sharing agreement, and the corresponding data set within a wider research project.

The results show considerable variability in Kappa values for certain variables, most notably "Type of Serve" in the intrareliability analysis (κ = 0.48 – 0.96 in Table 4a). While the overall values are acceptable, the discussion would be strengthened by briefly hypothesizing reasons for this high variability in specific matches or blocks. Was it due to a particular match context, a temporary lapse in applying the operational definition, or inherent subjectivity in classifying certain serve types?

A brief hypothesis of potential reasons for the variation of kappa values for type of serve was included in the discussion. To further emphasise its importance, a call for future research to investigate the variability of coding and potential causes was also included. Page 18-19, Lines 292 – 296.

The use of one secondary coder for establishing interreliability is a methodological limitation, albeit a common and often practical one. While the authors justify this within their context, the limitation section should more explicitly acknowledge how this choice might affect the generalizability of the interreliability findings compared to studies using multiple coders.

We appreciate and understand your comments here. As such, a statement regarding the highlighted limitation and its potential implications for future research has been included. Page 20, Lines 328-331

The operational definitions provided in Tables 1 and 2 are excellent. To further enhance the reproducibility and practical application of this work, the authors could consider including the complete coding template or a screenshot of the Nacsport window as Supporting Information (S1 File).

Firstly, thank you for the feedback regarding the operational definitions in Table 1 and 2. Due to this project laying the foundation for later studies and its position within a greater research project (with ongoing research being conducted), we have elected not to include a screenshot of Nacsport coding window. However, this suggestion will be taken into consideration for later publications.

**Responses to Reviewer 2**

I have carefully reviewed your work and appreciate your efforts to contribute to this field of research. While your research topic is of interest and has potential, I regret to inform you that I’m unable to propose acceptance of your manuscript in its current form. As the efficacy scale of volleyball skills was a basic instrument for the research, several methodological shortcomings and limitations were identified that require substantial revisions to ensure the paper's scientific validity. I believe that with careful attention to these areas, your manuscript has the potential to make a valuable contribution to the field. I would like to encourage you to revise your work and resubmit it for consideration.

We would like to thank you for taking the time to review our manuscript. It is pleasing that you see the potential in our work, and we will seek to address the comments provided to improve the manuscript.

In consideration of your comments below, we would just like to highlight that the efficacy scales used within this study align with efficacy scales that have been validated and implemented in the literature (Eom, 1989; Đurković, Marelić & Rešetar, 2009; Rabaz et al., 2013; Medeiros et al., 2017). In this regard the structure of the efficacy scales used within the current study mirror those of previous research, but there has been a conscious attempt to provide greater detail. Any significant changes would compromise the validity of the scale. We would also like to politely note that Reviewer 1 was complimentary of the detail in the operational definitions provided, so we are reluctant to make significant changes; however, we have responded to your comments where possible.

Serve Reception Modification: To eliminate the occurrence of a serve reception followed by a serve fault (Level 0), the serve reception scale must be truncated or shifted at least one level of efficacy. The remaining levels must be logically reversed with respect to serve efficacy levels (e.g., Level 1 Serve ↔Level 4 Serve Reception; Level 2 Serve ↔Level 3 Serve Reception, etc.).

Given the definitions provided for each of the skills, it is not possible for a serve fault (Level 0) to be followed by a serve receive, as a serve fault would mean that the rally has ended and no further skills can be performed. In addition, analysis of data evidences that a good serve receive is possible from a serve quantified as 1, 2 or 3 (i.e. the quality of the serve receive is not directly associated with the Serve but it is appreciated that a good Serve is more likely to lead to a poor quality Serve Receive). Whilst reversible efficacy scales have been utilised within the literature, such methods fail to appreciate the possibility of a serve being met by a serve receive of equal quality, something that is often observed in match play. In this regard, we do appreciate the sequential effect of such skills, and this is something we are seeking to give a greater level of appreciation in future work. We have made this a recommendation within the manuscript. Page 20 Lines 346-347.

Increased Differentiation in Serve Reception: Leaning on the precision of spatiotemporal recording systems like Nacsport Scout, the range for the non-scoring action (serve reception) should be expanded to more effectively distinguish among game situations created by the serve. Specifically, the lowest effective level of reception (presumably including "ball hit straight back to server" or "passed out from setter") should be separated into two distinct efficacy levels: Level 1: Ball is returned back directly to the serving team, with the potential to set up a counterattack (the receiving team must defend) and Level 2: Ball is served away from the setter, setting up a possible out-of-system attack (the receiving team still retains the right to attack). This is a significant distinction, as the following game dynamics are indeed different and need different efficacy ratings.

We appreciate your comment regarding the distinction of lower efficacy levels for the Serve Receive but given this study’s position in a broader project in which we are seeking to identify key skills within complexes, such changes are not plausible. As detailed previously such large scale changes would require the revalidation of a pre-established efficacy scale. The purpose of this paper was to identify the ability of analysts to reliably code the efficacy scores of individual skills within complexes. Future analysis would permit the identification of the frequency of serve receives that resulted in the transition to K5, but this was beyond the scope of this study. Although, we appreciate this is an interesting line of inquiry for future research. However, we have also made this a recommendation within the manuscript. Page 20 Line 344

Serve Scale Discrimination: Level 3 of the serve scale, which is currently combining an out of-system attack for the receiving team and a freeball for the serving team, must be fortified. Within competitive volleyball, the two scenarios are opposed opportunities for the serving team and must be seized by different assessment levels.

Thank you for your comment regarding the higher levels of the efficacy scale for the Serve. The efficacy scales have been informed by previous literature where a level 3 (or equivalent) refers to a serve that would typically result in an opposition disadvantage, which does include both out of system and freeball situations (Palao & Ortega, 2015; Medeiros et al., 2017; Šimac, Grgantov & Milić, 2017). By aligning our efficacy scales to those within the literature and previously used structures it provides greater opportunity for comparisons and discussion, not only within this study but within later research too. Similar to the above, we do appreciate your comments and as such have acknowledged these in the manuscript, particularly in relation to future research recommendations. Page 20 Lines 344-346.

The Block Evaluation Scale must contain greater definitional clarity across a number of different levels:

• Level 0 (Error/Point): Authors should specify the nature of error clearly. It needs clarification if Level 0 involves a blocking player's fault at the net or a block that causes the ball to touch the attacking side and subsequently fly out of the court.

• Level 1 (Clear Block): The definition of "clear block" ought to explicitly define its role as a known tactic (especially in women's volleyball) to intentionally guide the attack route towards a specific, specialised defence area or player.

•Level 3 (Block with Ball Remaining on Attacking Side): Improve this level. When a block has taken place, and the ball remains on the attacking side, the right to attack still exists for the team. This is a good plus, even though the subsequent first ball contact is not ideal, and the evaluation scale must reflect this tactical plus.

We had considered further clarifying the lower levels of the efficacy scale for the Block and are glad you agree with this. As such, further (minor) clarifications have been included to Table 2 (Page 8). However, such a change to the higher end of the efficacy scale would not align with the wider research project in which there is a distinct focus on these skills and the successful performance of them. As with previous comments regarding the efficacy scales, a statement has been included identifying the limitations of these scales and future research directions (Page 20 Lines 344- 346). Furthermore, we would like to note that within the literature efficacy scales implemented for the Block are typically more generic, referring to advantages, disadvantages and continuations, (Marcelino, Mesquita & Sampaio, 2011; Palao & Ortega, 2015; Klaričić, Grgantov & Jeleska, 2018) therefore, this is a first step towards more complex and nuanced definitions.

The current Setting evaluation scale presents formidable practical challenges to observers, even at the expense of system reliability. It necessitates setting a set as "in the general area of the hitter" to require the observer to judge simultaneously the spatiotemporal relationship between two dynamically moving points: the trajectory and appropriate speed of the ball, and the attacking centre of mass jumping (diagonal, vertical, or lateral movement). This very high degree of observer judgment provides the likelihood of measurement error and must be addressed either by the provision of more objective, quantifiable criteria or through the simplification of level definition. In my opinion, the evaluation of setting skill demands a more complex approach for the observer.

We agree with your concluding statement that Setting, and indeed all skills in volleyball, are complex in nature. Here, we would like to direct you to lines 265 – 278 in the discussion. Whilst the Kappa values for overall Skill Type and the types of some individual skills were above the acceptable threshold, Type of Set and Type of Spike were not. The drops in Kappa could be a result of camera angles, coder subjectivity, or the clarity of operational, however, understanding such decreases was beyond the aims of the research. In this regard, implementing such a complex analytical tool was beyond the scope of the broader research aim in which we were looking at the performance of skills in complexes. We acknowledge that there will be a high degree of subjectivity within the coding of this skill (and skill types), however, this is why we set out to assess the reliability of this measure, for which the intra- and inter-reliability of identifying the Set was above the acceptable threshold. Whilst the type of Set was below the threshold for inter-reliability, it was concluded that this element was beyond the scope of the broader research and was removed from later studies. Furthermore, during the design process of the coding window and the operational decisions, the accessibility for practitioners and applicability to practice were taken into consideration.

The manuscript lacks the operational definitions required for all skill types. These are the precursors to reproducibility and reliability.

•Spike/Attack Terminology: Spike classification is taxonomically inconsistent. Diagonal and line spikes are direction-based categories, while swipe and tip (or dink) are contact technique based categories. A swipe, for instance, can be executed as a diagonal or line attack. The authors must use a consistent classification criterion.

• Tempo Setting: Clear and unambiguous definitions for setting tempos need to be established, utilizing definite, established literature (Afonso et al., 2010) to ensure external validity and consistency with prior work in performance analysis.

We appreciate the request for further details regarding the types of skills implemented, however, we politely note that Reviewer 1 was complimentary of the detail of the operational definitions provided. We have acknowledged the potential reasoning behind the decrease in kappa and that further research is needed for the reliability of skill types (Pages 17-18 Lines 265-278). However, we also reiterate that the scope of the broader research project focuses on key skills across distinct complexes. Once these have been identified greater consideration can be given to the development of more detailed operational definitions to explore these areas in more detail. Given the focus of the study, we have elected to include the operational definitions as a supporting file rather than within the paper itself (Supporting File 1). Furthermore, we would like to note that given the position of this study within a larger research project, the element of skill types was removed as a variable for later analysis.

The study's stated objective was "to examine…, position,... and skill efficacy." The Methods section is inadequate because it provides no traceable information regarding the variable of position (e.g., player position, ball position, or rotation scheme by the team).

We have included clarification within the text regarding player position throughout the document.

Moreover, the paper does not include the mathematical formula or equation used in calculating the efficacy scores of skills, a necessary prerequisite to validate any bespoke performance analysis system. Not including this aspect makes it difficult for the peer reviewer and subsequent researchers to identify or reproduce the efficacy measure.

Regarding the mathematical formulas, none were used in this study. The scales within this study were all 5 levels and therefore did not require mathematical formulas. For the statistical processes, we would like to refer you to lines 181-195 on page 10.
